# Daily calving frequency and preterm calving is not associated with lunar cycle but preterm calving is associated with weather conditions in Japanese Black cows

**DOI:** 10.1371/journal.pone.0220255

**Published:** 2019-07-23

**Authors:** Yosuke Sasaki, Narumi Kitai, Mizuho Uematsu, Go Kitahara, Takeshi Osawa

**Affiliations:** 1 Department of Animal and Grassland Sciences, Faculty of Agriculture, University of Miyazaki, Miyazaki, Japan; 2 Center for Animal Disease Control, University of Miyazaki, Miyazaki, Japan; 3 Miyazaki Agricultural Mutual Aid Association, Miyazaki, Japan; 4 Department of Veterinary Sciences, Faculty of Agriculture, University of Miyazaki, Miyazaki, Japan; University of Florida, UNITED STATES

## Abstract

Several external factors including lunar cycle and weather conditions might be associated with calving conditions. Our objective here was to determine the effects of lunar cycle and weather conditions on calving frequency and the occurrence of preterm calving in Japanese Black cows. Calving records were obtained from 905 farms in Miyazaki Prefecture, Japan. Data were collected from 41,116 calvings. We conducted two studies: Study 1 investigated the effects of lunar cycle and weather conditions on daily calving frequency (DCF) with the observational unit of each day and Study 2 investigated those effects on the occurrence of preterm calving with the observational unit for each calving. Preterm calving was defined by whether or not a cow calving before 280 days of gestation, lower 10th percentile of gestation length of the collected data, and by whether or not a cow calving before 289 days of gestation, median of the gestation length. For Study 1, lunar cycle was not associated with DCF in all cows, in only primiparous cows and in only multiparous cows. As well as lunar cycle, weather conditions such as temperature, diurnal temperature variation, the temperature-humidity index, precipitation amount, barometric pressure, relative humidity and solar radiation, were also not associated with DCF. For Study 2, lunar cycle phases were not associated with the occurrence of preterm calving. However, preterm calving was associated with all of the weather conditions (P < 0.05) except for precipitation amounts and solar radiation. Temperature, the temperature-humidity index and relative humidity were positively associated with the occurrence of preterm calving. In contrast, diurnal temperature variations and barometric pressure were negatively associated with the occurrence of preterm calving. In conclusion, the lunar cycle was not associated with DCF and preterm calving, but the weather conditions were associated with preterm calving.

## Introduction

In bovine industry, it is crucial for herd managers to predict the onset of parturition because high perinatal calf mortality is related not only to economic losses but also to an animal welfare problem [1]. Herd managers, therefore, need to monitor parturition at a suitable time and take measures in preparation for unusual or abnormal conditions. In particular, large farms have several calving events every day, and it is useful to determine the factors affecting daily calving frequency (DCF) to improve management efficiency. As an indicator to predict DCF, several external factors including lunar cycle and weather conditions have been indicated to be possibly linked to calving conditions. In human, an increase on the percentage of deliveries in the full moon and the waning gibbous phases was found [2]. On the contrary, no difference was observed in the frequency of child births during various phase of lunar cycle [3]. A different analysis also demonstrated that there was no predictable influence of the lunar phases on the frequency of child births [4]. In sheep, similarly, the distribution of lambing dates did not differ among the various phases of the lunar cycle [5]. In cattle, one study reported that lunar cycles were associated with the frequency of spontaneous birth in 428 Holstein cows: thus, the frequency increased uniformly from the new moon to the full moon phase and then decreased [6]. However, since there has been contradictory findings among different reports in human and sheep, a much larger sample size is likely to be required to achieve an adequate statistical power.

From a different perspective, Troxel and Gadberry [7] found that weather patterns, such as a higher barometric pressure and lower temperature, increased the rate of calving in beef cows. However, information in this regard is still scarce so far. The Japanese Black (also known as “Wagyu”) is the most common beef cattle breed in Japan. These cattle are reared in uninsulated free stalls, and there is no difference in the air temperature between inside and outside the stall. Thus, lunar cycle and weather conditions might affect the calving frequency of these cows.

Stillbirth and dystocia have a significant impact on the productivity of beef industry. As well as calf losses, they are also associated with decreased subsequent reproductive performance of the dam [8]. Our previous research showed that the incidences of stillbirth and dystocia in Japanese Black cattle were more frequently observed as the gestation length was shorter [9]. Thus, it is vital to predict the onset of calving to have enough time to take care and assist increase viability of neonatal premature calves because many premature calves might at first appear to breathe normally but gradually develop respiratory distress over a period of several hours [10].

Our objective here was to clarify effect of lunar cycle and weather conditions on the rates of DCF and preterm calving in Japanese Black cows.

## Materials and methods

This research was carried out using data from farms in the suburban areas of Miyazaki city, located at 131° 24′ E longitude and 31° 56′ N latitude with an altitude of 9 m, in the southern part of Kyushu, Japan. In Miyazaki city the maximum, minimum temperatures and relative humidity in summer (July or August) were 31.3–32.1°C, 23.6–24.6°C and 75.6–79.7%, respectively, and the maximum, minimum temperatures and relative humidity in winter (January or February) were 12.8–13.6°C, 1.1–6.8°C and 58.2–73.4%, respectively, during the study period.

Calving data were available for 905 of the 1101 farms in the area. As described in detail previously [9], calving records were obtained from the database managed by the Miyazaki Prefecture Livestock Association, and 41,116 calvings in 15,378 animals between April 2006 and March 2010 were used for the analysis. The mean number of cows per farm was 18 (range 1–454). Cows were fed straw (rice, Italian ryegrass, or oat) twice daily to meet the requirements by Japanese feeding standard for beef cattle [11].

Weather data were collected from the database of the Japan Meteorological Agency for Miyazaki city. All of the 905 surveyed farms were located within a 20 km radius from the temperature measurement point. Their altitude above sea level was 46.9 ± 50.6 m (mean ± SD). The daily collected weather data were: mean temperature; maximum temperature; minimum temperature; precipitation amount; barometric pressure relative humidity and solar radiation. Diurnal temperature variation was calculated by subtracting the minimum from the maximum temperature. The temperature-humidity index (THI) was calculated as follows: THI = 0.8 × mean T + (mean RH/100) × (mean T– 14.4) + 46.4; where T is the temperature, and RH is the relative humidity [12]. In addition, accumulated measures of weather conditions over 2 days (from the day before to the day of calving: 0–1 days), 4 days (from three days before to the day of calving: 0–3 days) and 6 days (from five days before to the day of calving: 0–5 days) were calculated based on the assumption that sharp hormonal (e.g. progesterone, estrogen, prolactin, prostaglandins, etc.) and behavioral changes initiate one to five days before calving day [13,14].

Lunar data were also derived from data reported by the Japanese Meteorological Agency. The eight phases of the lunar cycle were categorized in accordance with a previous study [6]: new moon to waxing crescent (Lunar 1); waxing crescent to first quarter (Lunar 2), first quarter to waxing gibbous (Lunar 3), waxing gibbous to full moon (Lunar 4), full moon to waning gibbous (Lunar 5), waning gibbous to last quarter (Lunar 6), last quarter to waning crescent (Lunar 7), and waning crescent to new moon (Lunar 8).

This research was conducted in two studies, Study 1 and Study 2. Study 1 investigated the effect of lunar cycle and weather conditions on daily calving frequency (DCF). The observational unit was each day. The DCF was defined as the sum of calving events on each day. Each DCF was calculated from April 2006 to March 2010 by using the dataset of 41,116 calving records between April 2006 and March 2010 (there were 5,855 and 35,261 primiparous and multiparous cows, respectively). Because there was large variation in DCF among calving months (Table 1), an adjusted DCF value was calculated from the DCF on each day, subtracting the mean DCF value in the calving month and then used in the analysis. Study 2 investigated the effects of lunar cycle and weather conditions on the occurrence of preterm calving. The observational unit was calving and 41,116 calving records from Apr 2006 to Mar 2010 were used. The data sheet for Study 1 and Study 2 are given in the supporting information (S1 Table, S2 Table). Preterm calving was defined as whether a cow calving before 280 days of gestation, by the lower 10th percentile of gestation length of the collected data, and by whether a cow calving before 289 days of gestation: the median gestation length.

**Table 1:** Mean DCF values in each month.

		DCF ± SEM	
Month	All cows	primiparous cows	multiparous cows
January	27.5 ± 0.6	3.7 ± 0.2	23.7 ± 0.6
February	28.5 ± 0.6	4.2 ± 0.2	24.3 ± 0.6
March	30.0 ± 0.8	4.7 ± 0.2	25.3 ± 0.7
April	30.2 ± 0.6	4.9 ± 0.2	25.3 ± 0.6
May	30.5 ± 0.7	4.7 ± 0.2	25.8 ± 0.6
June	31.3 ± 0.7	4.4 ± 0.2	26.9 ± 0.6
July	30.6 ± 0.7	3.8 ± 0.2	26.8 ± 0.6
August	30.8 ± 0.7	3.7 ± 0.2	27.1 ± 0.6
September	25.4 ± 0.6	3.7 ± 0.2	21.8 ± 0.5
October	23.3 ± 0.6	3.1 ± 0.2	20.2 ± 0.6
November	25.1 ± 0.6	3.6 ± 0.2	21.5 ± 0.6
December	24.5 ± 0.6	3.6 ± 0.2	20.9 ± 0.5

Because the data used in this research were obtained from national and regional databases and no experiments were performed on live animals, Animal Care and Use Committee approval was not sought.

### Statistical analysis

The data were analyzed statistically with SAS Ver. 9.4 (SAS Institute Inc., Cary, NC, USA). For Study 1, a general linear model was applied. The dependent variable was the adjusted DCF for each day, and the independent variables were lunar cycle phase (Lunar 1 to Lunar 8) and daily weather data (mean temperature, maximum temperature, minimum temperature, diurnal temperature variation, THI, precipitation amount, barometric pressure, relative humidity and solar radiation). Each independent variable was included individually in the analysis. Daily weather data were used as continuous variables. Analyses were carried out by using all calving data for primiparous cows and calving data for multiparous cows. For Study 2, a mixed-effects logistic regression model was applied. The dependent variable was the occurrence of preterm calving, and the independent variables were the lunar cycle and weather data. Each independent variable was included individually in the analysis. Cow ID was included as a random effect in the statistical models. *P* values < 0.05 were considered to indicate statistically significant differences. Odds ratios and 95% confidence intervals were estimated for each factor.

## Results

### Study 1

**Fig 1:**
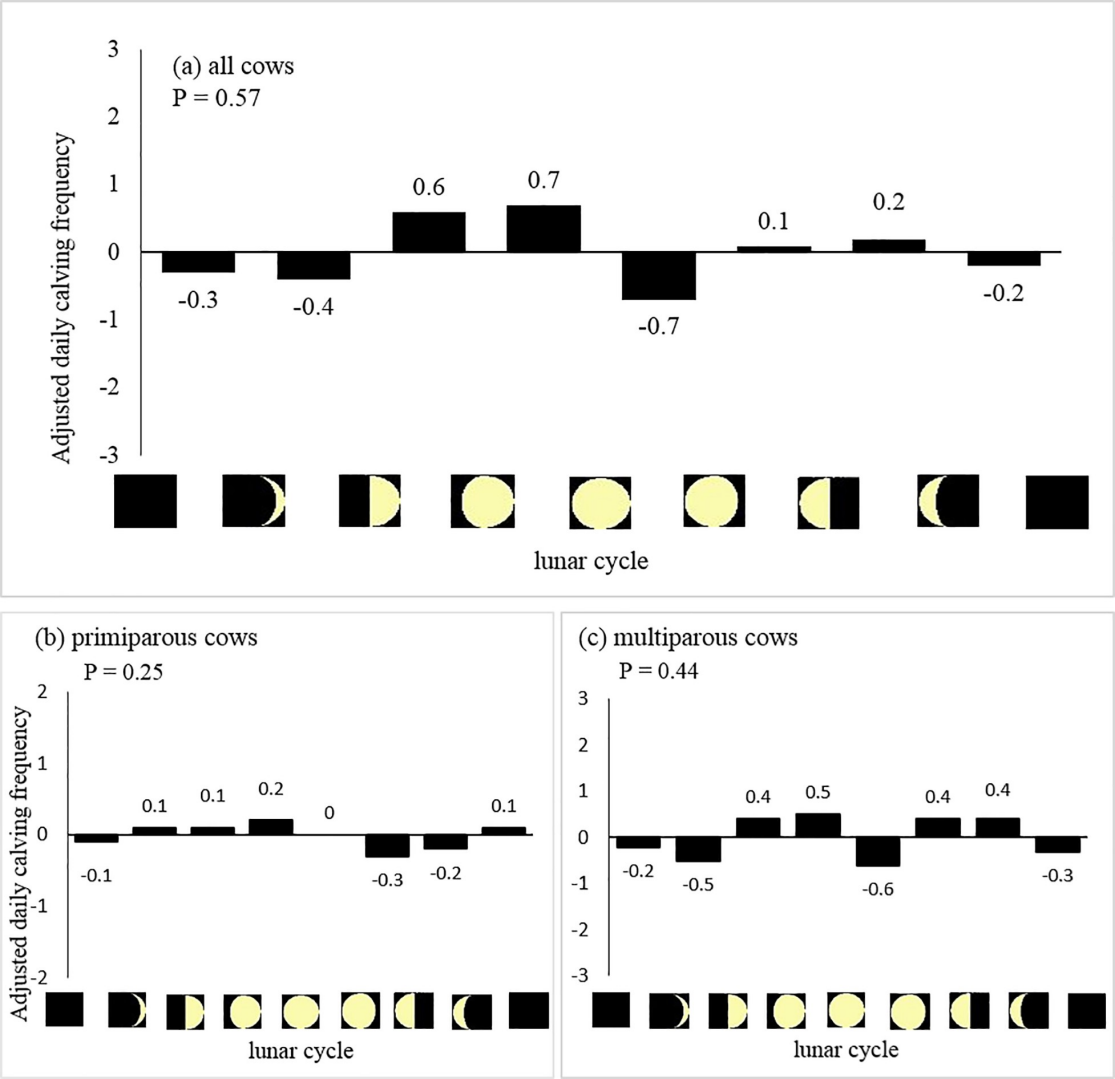
Distribution of adjusted DCF values by lunar cycle. (a) All cows; (b) Primiparous cows; and (c) Multiparous cows.

Mean DCF ± SD values in all cows, primiparous cows and multiparous cows were 28.1 ± 7.7, 4.0 ± 2.2 and 24.1 ± 6.9 calvings/day, respectively. Table 2 shows descriptive statistics of the weather conditions used for the analysis. The mean, maximum and minimum temperatures were positively associated with the adjusted DCF in all cows, but these associations were not significant statistically (Table 3). The other weather conditions, diurnal temperature variation, THI, precipitation amount, barometric pressure, relative humidity and solar radiation, were also not associated with the adjusted DCF. Likewise, adjusted DCF in primiparous or multiparous cows was not associated with weather conditions. Fig 1 shows the distribution of adjusted DCF values by lunar cycle. As well as weather conditions, lunar cycle phases were not associated with the adjusted DCF in any of the cow groups studied. No association was found between accumulated weather conditions for 2 days, 4 days or 6 days and adjusted DCF (S3 Table).

**Table 2:** Descriptive statistics of weather conditions used for the analysis.

Weather conditions	Unit	Mean	SD	Min	Max
Mean temperature	°C	17.9	7.2	2.3	31.2
Maximum temperature	°C	22.5	6.9	6.8	37.0
Minimum temperature	°C	13.8	7.9	-3.2	28.1
Diurnal temperature variation	°C	8.7	3.1	1.2	17.4
Temperature-Humidity Index		63.6	11.0	41.3	82.8
Precipitation amount	mm/day	6.9	19.9	0	280.5
Barometric pressure	hPa	1013	6.5	980.8	1030
Relative humidity	%	72.5	11.6	39	95
Solar radiation	MJ/m^2^	14.7	7.2	0.5	29.9

hPa: hecto-Pascals

**Table 3:** Association between weather conditions on the day of calving and adjusted DCF.

Weather conditions (WC)	Unit	Coefficient (± SEM) of WC	*P* value
Mean temperature	°C	0.0098 ± 0.0261	0.7083
Maximum temperature	°C	0.0077 ± 0.0273	0.7768
Minimum temperature	°C	0.0061 ± 0.0238	0.7978
Diurnal temperature variation	°C	– 0.0015 ± 0.0612	0.9810
Temperature-Humidity Index		0.0068 ± 0.0171	0.6878
Precipitation amount	mm/day	– 0.0028 ± 0.0094	0.7654
Barometric pressure	hPa	– 0.0166 ± 0.0288	0.5644
Relative humidity	%	0.0066 ± 0.0162	0.6829
Solar radiation	MJ/m^2^	0.0197 ± 0.0262	0.4525

### Study 2

**Fig 2:**
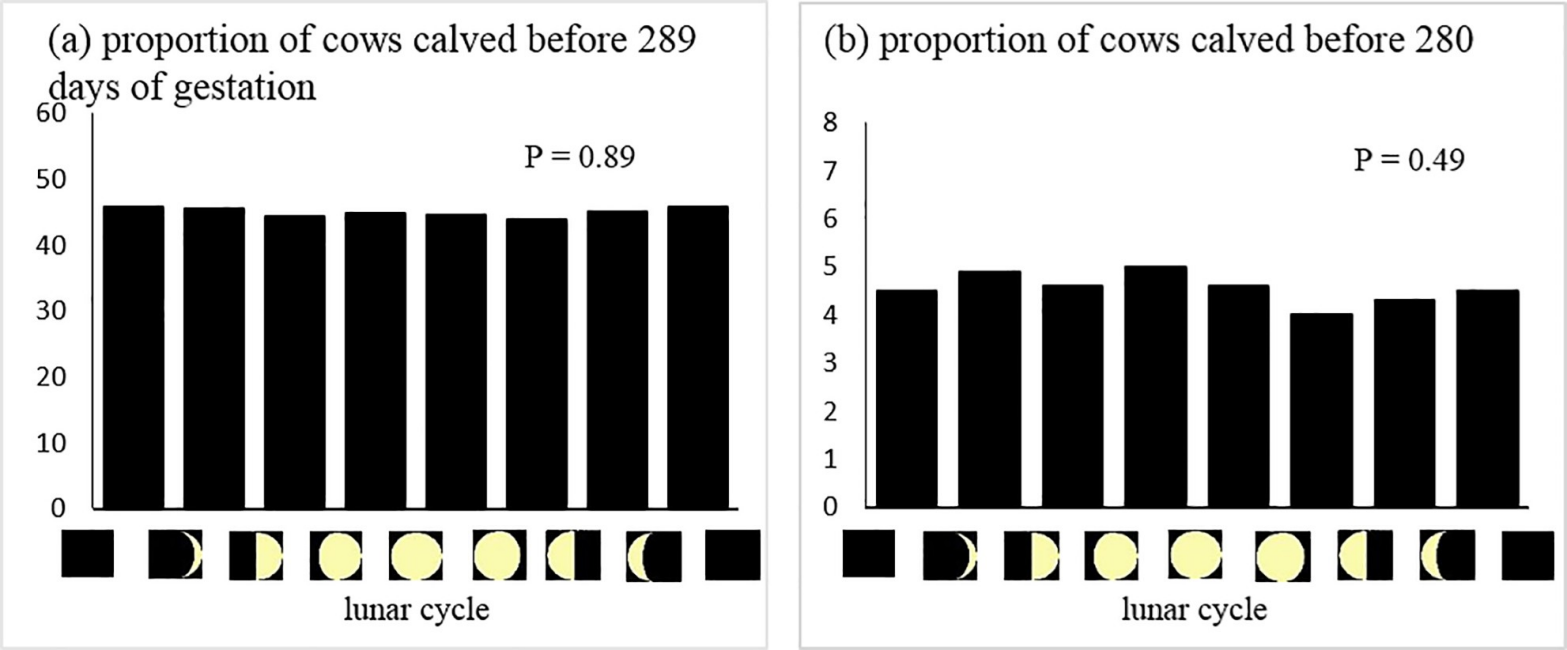
(a) Distribution of the proportion of cows calving before 289 days of gestation and (b) Proportion of cows calving before 280 days of gestation according to lunar cycle phase.

The mean gestation length and parity of 41,116 calving records was 289.6 ± 5.9 days and 4.9 ± 2.9, respectively. The occurrence of preterm calving was associated with all weather conditions except for precipitation amount and solar radiation (Table 4; *P* < 0.05). Mean, maximum and minimum temperature, THI and relative humidity were positively associated with the occurrence of preterm calving. In contrast, diurnal temperature variation and barometric pressure were negatively associated with the occurrence of preterm calving. Fig 2 shows the distribution of the occurrence of preterm calving by lunar cycle. Lunar cycle was not associated with the occurrence of preterm calving. No association was found between accumulated weather conditions for 2 days, 4 days or 6 days and the probability of preterm calving (S4 Table).

**Table 4:** Associations between weather conditions on the day of calving and the probability of preterm calving.

		Proportion of cows calving before 289 days of gestation		Proportion of cows calving before 280 days of gestation	
Weather conditions	Unit	Odd ratio	95% CI	Odd ratio	95% CI
Mean temperature	°C	1.024	1.021–1.027	1.021	1.014–1.028
Maximum temperature	°C	1.024	1.021–1.027	1.021	1.014–1.029
Minimum temperature	°C	1.022	1.019–1.025	1.019	1.013–1.025
Diurnal temperature variation	°C	0.977	0.971–0.984	0.982	0.967–0.997
Temperature-Humidity Index		1.016	1.014–1.018	1.013	1.009–1.018
Precipitation amount	mm/day	NS		NS	
Barometric pressure	hPa	0.985	0.982–0.988	0.986	0.980–0.994
Relative humidity	%	1.006	1.004–1.008	1.006	1.003–1.009
Solar radiation	MJ/m^2^	NS		NS	

## Discussion

Here, lunar cycle and weather conditions were investigated to test whether they were associated with DCF or preterm calving in Japanese Black cows. These variables were chosen because the lunar cycle has been reported to be associated with calving frequency in Holstein dairy cows [6] and with birth rates in humans [15], and weather conditions were also associated with calving frequency in beef cows [7]. To assess the effect of those variables on DCF and preterm calving, our research used two studies; one for each day level and the other for cow levels. Association of the variables of lunar cycle with DCF and preterm calving, and association of the variables of weather conditions with DCF and preterm calving are discussed in the following paragraphs separately, for convenience.

There was no effect of lunar cycle on DCF or the occurrence of preterm calving in Japanese Black cows. This finding disagrees with a previous study conducted in Hokkaido, Japan, in which calving frequency in Holstein dairy cows increased uniformly from the new moon to the full moon phase [6]. Although the biological reason for this difference is unknown, the effects of lunar cycle on calving condition may be different among breeds or regions. The previous study was conducted in Hokkaido located at a latitude of 43° N, whereas our research was carried out in Miyazaki located at 33° N. This difference of 10° of latitude leads to different lengths of daylight and moonlight, which might have had a considerable influence on the results of the previous and our observations. There are many possible factors that could explain the difference. However, our research was based on a large dataset (more than 40,000 calvings) collected from more than 900 farms over several years and therefore, are scientifically valid and robust. In human, relationship of obstetric delivery with lunar cycle has been studied and discussed. Although some studies showed a positive association [2,15], most of the studies demonstrated no association between them [3,16–21]. Recently, Marco-Gracia performed a retrospective cohort analysis on 23,689 spontaneous deliveries within the home for 1484 lunar cycles between the years 1810 and 1929 in rural Spanish villages. The author showed that there was no pattern with which to link lunar phases with the frequency of births in women who did not have electricity in their daily lives and concluded that the belief in the relationship between lunar phases and human births is simply a myth [22].

We found that high temperature and THI conditions might increase the occurrence of preterm calving. A similar result was reported indicating that a high temperature on the days before parturition led to a shortened gestation length in dairy and beef cows [7,23]. In sheep, heat stressed by exposure to a temperature of 48°C for 75 min increased serum oxytocin and antidiuretic hormone levels [24]. Increased oxytocin causes the release of prostaglandin F_2α_ that leads to an early onset of parturition. In human, proteins of the heat shock 70 family have been associated with premature rupture of fetal membranes and premature delivery and thus with premature babies [25]. In contrast to temperature, diurnal temperature variations had a negative effect on the occurrence of preterm calving in this study. This is in disagreement with a previous study reporting that diurnal temperature range decreased gestation length and increased birth rates [9,26]. Sudden changes in the temperature were associated with the release of inflammatory mediators by mast cells, and immune and inflammatory processes have been considered as important underlying mechanisms of preterm birth [27–29]. However, the relationship between diurnal temperature variations and preterm calving could be an inverse result of that for temperature because diurnal temperature variation was negatively correlated with ambient temperature during the study period in Miyazaki. Nevertheless, further study is needed to clarify associations between diurnal temperature variation and preterm birth. Premature birth was reported to be related with stillbirth and dystocia [9] and decreased reproductive performance [8]. Therefore, it is important to take care of premature birth to assist increase viability of neonatal calves.

Here we found that barometric pressure conditions were not associated with DCF. No association between barometric pressure and the onset of parturition was detected in two previous studies involving human births in two different hospitals [19,30]. However, low barometric pressure increased the occurrence of preterm calving in cows in the present research. In another study in humans, rupture and premature rupture of the fetal membranes occurred on days when the barometric pressure was below the average of the preceding 8 years [31]. Changes in barometric pressure has been suggested to trigger parturition in cattle that are near term gestation by influencing adrenal secretion of glucocorticoids [32]. Preterm birth is a complex syndrome initiated by multiple causes and the etiology of preterm birth remains poorly understood [33], and therefore, further study is warranted to identify possible underlying pathways in the association between weather conditions and preterm calving.

## Conclusions

We conclude that the lunar cycle was not associated with calving frequency or preterm calving in Japanese Black cattle, but weather conditions such as high temperature and THI increased the occurrence of preterm calving. However, it should be noted that this research was not a controlled experiment, but an observational study using records from commercial farms. Thus, these findings should be interpreted only as associations, not as indicators of biological causation. Additionally, the lack of associations found in this study might have been masked by other factors both on an individual cow and herd levels.

## Supporting information

S1 Table Data sheet for Study 1. (XLSX)

S2 Table Data sheet for Study 2. (XLSX)

S3 Table Association between accumulated weather conditions for 2 days, 4 days and 6 days, and adjusted DCF. (XLSX)

S4 Table Associations between accumulated weather conditions for 2 days, 4 days and 6 days, and the probability of preterm calving. (XLSX)
